# The Role of Magnetic Resonance Imaging in Cardiomyopathies in the Light of New Guidelines: A Focus on Tissue Mapping

**DOI:** 10.3390/jcm13092621

**Published:** 2024-04-29

**Authors:** Cinzia Forleo, Maria Cristina Carella, Paolo Basile, Donato Mandunzio, Giulia Greco, Gianluigi Napoli, Eugenio Carulli, Marco Maria Dicorato, Ilaria Dentamaro, Vincenzo Ezio Santobuono, Riccardo Memeo, Michele Davide Latorre, Andrea Baggiano, Saima Mushtaq, Marco Matteo Ciccone, Gianluca Pontone, Andrea Igoren Guaricci

**Affiliations:** 1 University Cardiologic Unit, Interdisciplinary Department of Medicine, Polyclinic University Hospital, 70124 Bari, Italy; cinzia.forleo@uniba.it (C.F.); m.c.carella92@gmail.com (M.C.C.); paolo.basile@uniba.it (P.B.); donato.mandunzio@gmail.com (D.M.); giulia-greco@libero.it (G.G.); gianluiginapoli@gmail.com (G.N.); e.carulli93@gmail.com (E.C.); mm.dicorato@gmail.com (M.M.D.); ilaria.dentamaro@gmail.com (I.D.); vincenzoezio.santobuono@uniba.it (V.E.S.); dottor.riccardomemeo@gmail.com (R.M.); latorre.michele.d@gmail.com (M.D.L.); marcomatteo.ciccone@uniba.it (M.M.C.); 2 Department of Perioperative Cardiology and Cardiovascular Imaging, Centro Cardiologico Monzino, IRCCS, 20138 Milan, Italy; andrea.baggiano@cardiologicomonzino.it (A.B.); saima.mushtaq@cardiologicomonzino.it (S.M.); gianluca.pontone@cardiologicomonzino.it (G.P.); 3 Department of Biomedical, Surgical and Dental Sciences, University of Milan, 20138 Milan, Italy

**Keywords:** cardiomyopathies, tissue mapping, cardiac magnetic resonance, advanced diagnostic techniques, non-dilated left ventricular cardiomyopathy, cardiogenetic

## Abstract

Cardiomyopathies (CMPs) are a group of myocardial disorders that are characterized by structural and functional abnormalities of the heart muscle. These abnormalities occur in the absence of coronary artery disease (CAD), hypertension, valvular disease, and congenital heart disease. CMPs are an increasingly important topic in the field of cardiovascular diseases due to the complexity of their diagnosis and management. In 2023, the ESC guidelines on cardiomyopathies were first published, marking significant progress in the field. The growth of techniques such as cardiac magnetic resonance imaging (CMR) and genetics has been fueled by the development of multimodal imaging approaches. For the diagnosis of CMPs, a multimodal imaging approach, including CMR, is recommended. CMR has become the standard for non-invasive analysis of cardiac morphology and myocardial function. This document provides an overview of the role of CMR in CMPs, with a focus on tissue mapping. CMR enables the characterization of myocardial tissues and the assessment of cardiac functions. CMR sequences and techniques, such as late gadolinium enhancement (LGE) and parametric mapping, provide detailed information on tissue composition, fibrosis, edema, and myocardial perfusion. These techniques offer valuable insights for early diagnosis, prognostic evaluation, and therapeutic guidance of CMPs. The use of quantitative CMR markers enables personalized treatment plans, improving overall patient outcomes. This review aims to serve as a guide for the use of these new tools in clinical practice.

## 1. Introduction

Cardiomyopathies (CMPs) are a group of myocardial disorders characterized by structural and functional abnormalities of the heart muscle, in the absence of coronary artery disease (CAD), hypertension, valvular disease, and congenital heart disease (CHD) [1,2]. CMPs are an increasingly important topic in the field of cardiovascular disease, not least because of the complexity of their diagnosis and management. This is demonstrated by the fact that the European Society of Cardiology (ESC) guidelines on cardiomyopathies were published for the first time in 2023 and are not just an update of previous recommendations, but a first edition. The boom in this field has been made possible by the development of two techniques in particular: cardiac magnetic resonance (CMR) imaging and genetics [3,4].

Imaging techniques have evolved over the years, allowing a better understanding of these diseases [5,6]. For this reason, a multimodal imaging approach, including ultrasound-based techniques, CMR imaging, computed tomography (CT), positron emission tomography (PET), and scintigraphy, is actually recommended in their diagnostic workup [2,7,8,9]. Among all these techniques, CMR has become over the years the gold standard for non-invasive assessment of cardiac morphology, function, and myocardial tissue characterization [10,11,12]. With this document we aim to provide an overview of CMR imaging findings in CMPs that may be of practical use to clinicians.

## 2. CMR-Based Sequences and Techniques

### 2.1. Morphology and Function

Through the application of non-gated balanced steady-state free precession (b-SSFP) sequences and the acquisition of cine images with high spatial and temporal resolution, CMR represents the gold standard imaging modality for the quantification of cardiac chamber size, volume, mass, and global or regional function, with a much greater morphological characterization compared with echocardiography [13,14,15,16]. Furthermore, the deformation (strain and strain rate) of myocardial segments can be measured through post-processing analysis of b-SSFP sequences with feature-tracking (FT) technology in order to assess early changes in myocardial mechanics and function [17,18].

### 2.2. Tissue Characterization

More recently, advanced non-invasive tissue characterization can be achieved using appropriate CMR sequences like late gadolinium enhancement (LGE) imaging and parametric mapping techniques.

LGE T1-weighted (T1-W) images are based on the typical extracellular distribution kinetic of a paramagnetic gadolinium-based contrast agent (GBCA), which is normally washed away in 10–20 min. With damaged myocardial tissue, there is a larger extracellular space and more enhancement after gadolinium, allowing MRI to detect myocardial infarcts as well as nonischemic necrosis, fibrosis, and amyloid deposition. T2-weighted (T2-W) short tau inversion recovery (STIR) images, instead, are effective in detecting water accumulation due to inflammatory extracellular edema [13,15,19].

CMR parametric mapping uses several advanced imaging techniques, allowing for a quantitative assessment of myocardial tissue properties. Unlike traditional imaging methods, such as echocardiography, cardiac MRI mapping provides detailed information on tissue composition, fibrosis, edema, perfusion, and contractility [20]. This modality integrates data deriving from T1, T2, and T2* (star) mapping, as well as from extracellular volume fraction (ECV) quantification, so that any change in myocardial composition can be visualized, offering insights into pathological processes previously inaccessible, except through histological examinations [15,21].

CMR sequences characteristics and applications are summarized in Table 1.

## 3. Dilated Cardiomyopathy

Non-ischemic dilated cardiomyopathy (DCM) is a condition characterized by dilation and weakening of the heart muscle, resulting in a reduced pumping capacity of the ventricles [2]. The dilation is not caused by reduced blood supply to the heart muscle or abnormal stress conditions but by other factors such as viral infections, autoimmune reactions, toxins, alcohol abuse, genetic causes, or unknown factors [22].

It is important to note the superiority of magnetic resonance imaging over echocardiography in accurately quantifying the volume of the cardiac chambers and important parameters, such as ejection fraction, for therapeutic decisions in this patient setting [23].

The 2023 guidelines on cardiomyopathies establish, for the first time, that the presence of late LGE on MRI in patients carrying variants in genes associated with high arrhythmic risk predicts an increased risk of sudden cardiac death [2]. This may indicate the need for a prophylactic implantable cardioverter defibrillator in the presence of other variables or when certain risk score cut-offs are reached. Diffuse interstitial fibrosis can be detected by T1 mapping, whereas irreversible fibrotic replacement corresponds to the presence of LGE [24,25]. Although each scanner has its own reference values, the normal range of native T1 is 900–1035 ms at 1.5 T, with a higher value generally associated with the presence of diffuse fibrosis. An observational study demonstrated that the existence of anomalies in the native myocardial T1 relaxation times might serve as an even more effective and autonomous indicator of unfavorable prognosis among individuals with DCM [26]. Other investigations have established a link between ECV and cardiac incidents: in a cohort of patients diagnosed with non-ischemic DCM, the degree of irregularity determined through ECV mapping forecasts a progressively heightened susceptibility to heart failure consequences [27]. The study found a strong correlation between ECV and major adverse cardiac events (MACE) across various anatomical locations, with the most significant association observed in the anteroseptal region [27,28,29]. Furthermore, ECV exhibited an additional and incremental predictive relationship with MACE when compared to native T1, the presence of LGE, and the extent of LGE mass [27,28,29]. In a recent study, the potential predictive value of quantitative CMR features for MACEs in patients diagnosed with DCM was investigated. The study found that patients who experienced heart failure or arrhythmia-related events had significantly higher levels of both native T1 and ECV compared to non-ischemic DCM patients without MACE [28].

T2 mapping is highly effective in identifying early stage DCM, especially when myocardial morphology is challenging to distinguish from athletic myocardial adaptation [30]. In DCM, higher values are reported than in normal subjects (62.9 ± 5.7 ms vs. 55.4 ± 3.5 in controls) [31]. It also holds prognostic value: the shortening of myocardial T2 relaxation time may indicate which patients are more likely to undergo left ventricular reverse remodeling during treatment [32].

Incorporating these quantitative CMR markers of diffuse interstitial disease into clinical practice allows for the customization of therapeutic approaches, including the consideration of implantable cardioverter defibrillator (ICD) placement and cardiac resynchronization therapy (CRT), as previously mentioned [33]. The use of these markers can help tailor treatment plans to individual patients, improving their overall outcomes.

The idea that the presence and size of LGE is only the “tip of the iceberg” and that it is necessary to characterize diffuse myocardial fibrosis to improve risk stratification in DCM patients is gaining ground [27].

## 4. Non-Dilated Left Ventricular Cardiomyopathy

Since 2023, the ESC has introduced a new category of cardiomyopathies named non-dilated left ventricular cardiomyopathy (NDLVC). This new classification includes cases where there are abnormalities in wall kinetics or scar tissue in the left ventricle without dilation [2]. This category may include patients in the early stage of dilated cardiomyopathy (DCM) or those who were previously classified within the spectrum of non-dilated hypokinetic cardiomyopathies, as well as all forms of arrhythmogenic involvement of the left ventricle that were once grouped under the umbrella term of arrhythmogenic cardiomyopathy (ACM).

Few specific studies on the subject have been published, and the prevalence, diagnosis, and management of NDLVC remain undetermined [34,35,36,37].

In this context, it is important to use MRI to detect all areas of myocardial scar or fibroadipose replacement that echocardiography cannot highlight, given the definition of NDLVC. The guidelines themselves highlight the importance of quantifying and describing the LGE pattern for suspecting a specific genetic etiology: in dystrophinopathies, LGE typically manifests as extensive inferolateral patterns, while LMNA carriers commonly exhibit mid-wall septal LGE; DSP and FLNC variant carriers often present with a ring-like LGE pattern [2,38]. Little is still known about the role of mapping in this patient category, although it is presumed to be similar to that implied for individuals with DCM. The detection of myocardial edema may suggest an inflammatory or myocarditic origin [2].

MRI will be crucial in the early identification of individuals affected by NDLVC and their family members. It could aid in prognostic stratification, although initial data show no significant difference in the incidence of cardiac events between NDLVC with reduced ejection fraction and individuals with classic DCM [37]. Therefore, both categories require equal attention in follow-up and management [37].

## 5. Hypertrophic Cardiomyopathy

Hypertrophic cardiomyopathy (HCM), characterized by excessive myocardial wall thickening or mass and impaired diastolic filling that is not solely explained by abnormal loading conditions [1,2], benefits from the ability of cardiac MRI to accurately measure myocardial thickness, ECV, and regional strain. 

HCM is often correlated with the appearance of diffuse myocardial fibrosis, detected by late gadolinium enhancement (LGE). Elevated native T1 values have been identified not only in regions corresponding to LGE, but also even in the absence of regionally apparent LGE and hemodynamic obstruction [39,40]. This implies that native T1 could identify tissue abnormalities prior to the development of fibrosis detected by LGE [39,40]. Individuals with HCM exhibit heightened interstitial fibrosis within the hypertrophied sections, even in the absence of late gadolinium enhancement (LGE). Additionally, elevated T1 and ECV measurements were linked to the left ventricular mass index across the entire HCM patient group [41]. 

The severity of left ventricular hypertrophy is expressed to a greater extent by the prolongation of T2 time than T1 time [42]. T2 time is also used to distinguish compensatory hypertrophy in athletes, with a greater increase in T2 observed in patients with HCM [43].

**Figure 1:**
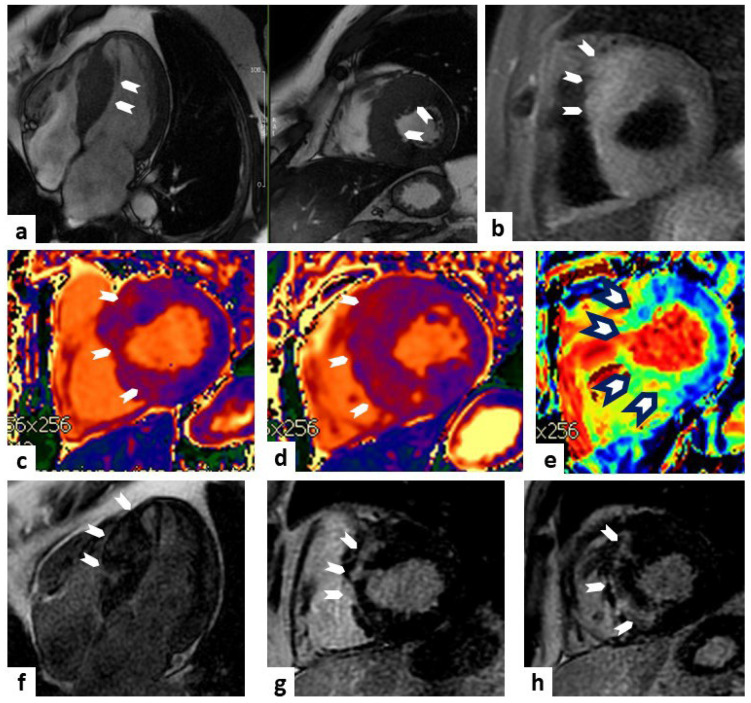
Cardiac MRI of hypertrophic cardiomyopathy (HCM). A case of a 38-year-old male with a history of premature ventricular beats. Panel (**a**) (SSFP sequences) represents a four-chamber and short axis views (top left panel and top middle panel, respectively) showing a marked and asymmetric hypertrophy of the interventricular septum (white arrowheads). Panel (**b**) (TIR t2 sequence) represents a short-axis view showing a higher signal al the level of the septum (white arrowheads) compared to the remote myocardium. Panels (**c**,**d**) represent increased values on T1 mapping sequences at the level of the basal and middle septum (white arrowheads), respectively. Panel (**e**) represents increased values on extracellular volume (ECV) sequence at the level of middle septum (white arrowheads black outlines). Panels (**f**–**h**) (LGE sequences) display a non-ischemic pattern of hyperenhancement at the level of septum (white arrowheads). Taken together, these findings are diagnostic of asymmetric HCM.

MRI is considered the gold standard in the differential diagnosis of different forms of cardiomyopathy with hypertrophic phenotype (Figure 1).

T1 and T2 mapping helps identify areas of fibrosis, edema, and inflammation, aiding risk stratification and treatment planning. Quantitative cardiac MRI data contribute to a better understanding of disease progression and guide interventions, such as septal reduction therapies.

## 6. Cardiac Amyloidosis

Amyloidosis is a rare group of infiltrative diseases caused by protein misfolding and the subsequent extracellular deposition of the abnormal proteins (amyloid fibrils) in various tissues and organs, leading to gradual organ failure [44]. Cardiac amyloidosis (CA) is a serious and underdiagnosed condition, mainly caused by deposition of two precursor proteins in myocardial tissue: transthyretin amyloid (ATTR) and monoclonal light chain amyloid (AL). AL amyloidosis, due to production of monoclonal light chains by a small B-cell clone, has long been considered the most common form of systemic amyloidosis, with cardiac involvements in 50–70% of cases [45]. However, recent studies show that the prevalence of the different forms may vary considerably [46]. ATTR amyloidosis is caused by the deposition of misfolding transthyretin, a protein synthesized in the liver, normally responsible for the transport of retinol and thyroxin binding protein [44]. The non-hereditary wild type form (ATTRwt) has late onset and involves exclusively the heart, while the hereditary form (ATTRh) affects younger patients and is typically associated with polyneuropathy [47,48].

Endomyocardial biopsy (EMB) is the gold standard for the diagnosis of CA: amyloid fibrils are recognized by their characteristic apple green birefringence with Congo Red coloring and observation under a polarized light microscope [49]. The limitations of EMB are errors in tissue processing and availability of expertise. Imaging offers a noninvasive alternative to evaluate the whole heart: long thought to be of limited use in this pathology, CMR has been shown to be a reproducible and sensitive imaging modality that plays a key role in the diagnostic algorithm of CA and helps to assess the progression or regression of cardiac involvement during the course of therapy [46,47,48].

CMR allows for the assessment of cardiac morphology and function using cine images obtained with SSFP sequences acquired in long- and short-axis planes covering the left ventricle (LV) [47,48]. The most common phenotype of ATTR patients is asymmetrical LV hypertrophy, while symmetrical and concentric LV hypertrophy is present in 68% of AL patients [45].

LGE has a characteristic distribution and is correlated with the degree of the LV infiltration: in early stages it is fuzzy and focal; in advanced stages it is diffuse, subendocardial, transmural, or binary, with greater involvement of basal segments than the apical one [50]. QALE (query amyloid late enhancement) score can also quantify the degree of LGE: the total score ranges from 0 (no LGE) to 18 (global transmural LV LGE and right ventricle (RV) involvement) [51]. 

The LGE pattern is also associated with different kinetics in the clearance of gadolinium in the blood and myocardium [52]. Unfortunately, LGE should be administered with caution in patients with moderate to severe renal disease (eGFR < 30 mL/min), which represents a substantial number of subjects with CA, particularly AL CA, because of renal infiltration of the AL amyloid [44,53]. 

In patients where GBCA is contraindicated, native T1 mapping (nT1) and ECV have emerged as quantitative techniques to track myocardial amyloid infiltration and monitor disease severity [45]. ECV and nT1 are typically increased in patients with CA; nT1 is frequently higher in the early stages of CA prior to the development of detectable LGE and biventricular thickening [44].

Baggiano et al. show that nT1 has excellent diagnostic accuracy in an overall population of patients with clinical suspicion of amyloidosis, potentially supporting the routine use of non-contrast CMR in this setting: in a subject with myocardial native T1 > 1164 ms (z-score, 3.5), cardiac amyloidosis can be diagnosed with very high diagnostic accuracy (PPV 98%); in a subject with native myocardial T1 < 1036 ms (z-score, 0.4), CA can be excluded [54]. A recent single-center prospective study involving 221 patients with AL CA demonstrated how nT1 can track response to chemotherapy treatment in this disease: patients who decreased native T1 had a good prognosis and better hematologic response to treatment, in contrast to patients whose native T1 increased or remained stable [55]. This study therefore confirms and paves the way on the role of native T1, not only in the diagnosis of amyloidosis but also as an accurate marker of response to treatment and correlated with survival [55].

**Figure 2:**
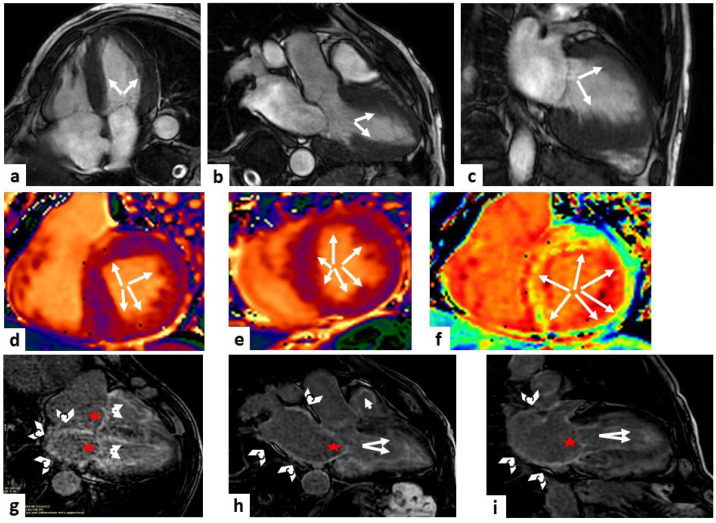
Cardiac amyloidosis. A case of a 79-year-old man, with magnetic resonance imaging (MRI) showing a hypertrophic (white arrows) left ventricle (LV) of 17 mm in the steady-state free precession (SSFP) sequences (panels (**a**–**c**) show four-chamber, three-chamber, and two-chamber views, respectively). Mid panels show diffuse high values at T1 mapping sequences, represented by the orange tonality as indicated by the white arrows (panels (**d**,**e**) at the level of the basal and mid-LV short-axis views, respectively) and diffuse high values at extracellular volume (ECV) sequence, represented by the yellow tonality as indicated by the white arrows (panel (**f**) at the level of the basal LV short-axis view). Late gadolinium enhancement (LGE) sequences showed a non-ischemic enhancement pattern in both ventricles (white arrows), a hyperenhancement of the atrio-ventricular valves (red stars), of the left atrium wall and the interatrial septum (white arrowheads) (panels (**g**–**i**) show four-chamber, three-chamber, and two-chamber views, respectively). All these findings are diagnostic of cardiac amyloidosis.

Postcontrast T1 mapping, which can be incorporated into standard LGE-CMR protocols, is useful to compute an ECV increase caused by amyloid infiltration (Figure 2). Recent studies demonstrated that high levels of ECV (ECV > 0.40%) can help to diagnose CA early on and are a prognostic sign in both ATTR and AL amyloidosis [56,57].

T2 mapping is a noncontrast sequence and, as compared to T1 mapping, is more specific to detect myocardial oedema. T2 mapping values are increased in patients with both forms of CA, with a greater prevalence in AL than in ATTR.

## 7. Anderson–Fabry Disease

Anderson–Fabry disease (AFD) is an X-linked lysosomal storage disorder, associated with the mutation in the α-galactosidase gene, which results in the deposition of glycosphingolipids in several organs and tissues [58]. Cardiac involvement, which occurs in approximately 70% of cases, represents the most prevalent cause of death in these patients [58]. It involves the accumulation of sphingolipids in all cardiac tissues: in the cardiomyocytes, leading to concentric hypertrophy and myocardial dysfunction; at the valve level, causing structural and functional changes (most commonly mitral and aortic regurgitation); in the conduction tissue, causing electrophysiological remodeling that may lead to arrhythmias; and finally at the endothelial level, where inflammatory and fibrotic mechanisms may lead to endothelial dysfunction and coronary microvascular ischemia [59,60].

**Figure 3:**
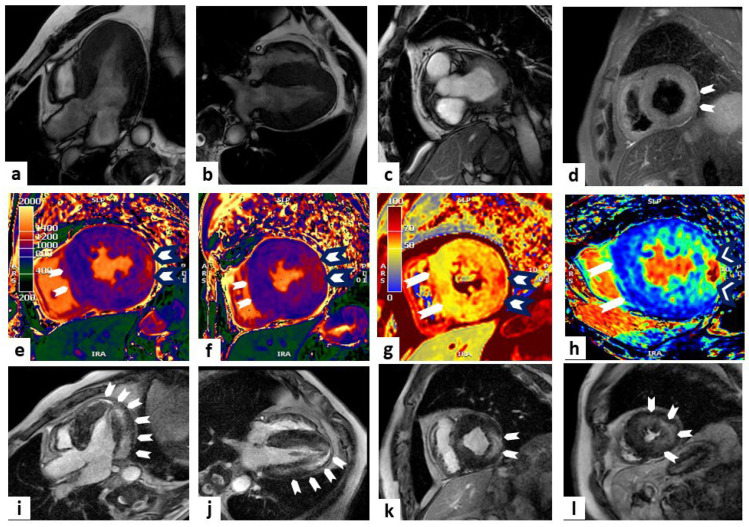
Anderson–Fabry disease. One case involves a 67-year-old man who was diagnosed with Anderson–Fabry disease through genetic testing during family screening (because of his affected brother). He remains completely asymptomatic. Cardiac magnetic resonance (CMR) imaging showed concentric LV hypertrophy with a mild reduction in ejection fraction (EF) in steady-state free precession (SSFP) sequences (panels (**a**–**c**) show three-chamber, four-chamber, and short-axis views at the basal level, respectively). Panel (**d**) shows oedema (white arrowheads) at the level of the mid-LV short-axis view (T2-weighted sequence). Panels (**e**,**f**) display T1 mapping sequences with a low value in the septum (white arrowheads) and higher values in the lateral wall (white arrowheads with black outlines) at the level of LV papillary muscles and the mid-LV short-axis view, respectively. Panels (**g**,**h**) display T2 mapping and extracellular volume (ECV) sequences, respectively, with low values in the septum (white arrowheads) and higher values in the postero-lateral wall (white arrowheads with black outlines) at the level of the mid-LV short-axis view. Late gadolinium enhancement (LGE) sequences display a non-ischemic hyperenhancement pattern (white arrowheads) at the level of the lateral wall (panels (**i**–**l**) show three-chamber, four-chamber, mid-LV short-axis, and apical LV views, respectively).

CMR represents the predominant non-invasive imaging modality in the early diagnosis and staging of AFD, as it combines the assessment of cardiac involvement and the characterization of tissue abnormalities [61] (Figure 3).

The most common morphological finding is a concentric hypertrophy of LV and of papillary muscles, usually associated with hypertrophy of RV [62,63]. Another typical finding is the presence of LGE, described as myocardial fibrosis areas, in the basal inferolateral mid-wall of the LV with sub-endocardial sparing, found in up to 50% of subjects [58].

Intracellular accumulation of glycosphingolipids causes a reduction in nT1, especially at the basal septum, even at the early stage, that often precedes ventricular hypertrophy and may therefore be useful as a marker for early initiation of enzyme replacement therapy [64]. This makes this method useful for the early identification of cardiac involvement, before the morphological and functional alterations typical of the full-blown phases of the disease [65]. 

However, it has been shown that, at a more advanced stage of the disease, when inflammation is active and there is a recall of lymphocytes, there is a pseudo-normalization of T1 time that could mislead the clinician [66]. This phenomenon has led experts to propose a three-phase model of AFD: in the initial phase, termed accumulation, there is a reduction in T1 mapping; in the second phase, termed inflammatory, ventricular hypertrophy begins to manifest and T1 mapping can be within a normal range; finally, there is the irreversible terminal phase, with the development of fibrosis and evidence of LGE [66,67].

Regarding T2-weighted (T2W) images and T2 mapping sequences, these play a crucial role in assessing overall myocardial inflammation, particularly during the initial phases of the disease [68].

In the research conducted by Frustaci et al., myocardial edema associated with AFD was identified in the basal antero-septal wall (70%) and occasionally in the antero-lateral wall, showing a sporadic distribution within the mid-wall region [69]. Furthermore, they demonstrated that myocardial edema increased simultaneously with LV hypertrophy in 31% of the patients [69]. Conversely, Perry and colleagues, in their study, observed an elevated signal in the basal inferior-lateral area and a reduction in T2 relaxation time proportional to the reduction in LV mass and thus to the response to therapy [65]. Furthermore, Augusto et al. established a correlation between T2 mapping values and troponin levels, implying that cardiac involvement in AFD leads to a persistent inflammatory cardiomyopathy: in stages where the disease is more active and myocardial damage is ongoing, T2 mapping increases in proportion to the degree of oedema and hs-TnI [68]. 

As a result, comprehensive cardiovascular magnetic resonance (CMR) assessment should always include T1 and T2 mapping sequences to effectively identify and monitor AFD in both suspected and confirmed cases.

## 8. Arrhythmogenic Right Ventricular Cardiomyopathy

According to the latest ESC guidelines on CMPs, arrhythmogenic right ventricular cardiomyopathy (ARVC) is defined as the presence of predominantly RV dilatation and/or dysfunction in the presence of histological involvement and/or electrocardiographic abnormalities, based on the revised International Task Force (ITF) criteria for the diagnosis of ARVC published by Marcus et al. in 2010 [2,70]. Recently, the identification of two other possible phenotypes (biventricular and left-dominant) has led to the proposed term “arrhythmogenic cardiomyopathy” (ACM) and to new diagnostic criteria that include LV involvement, but they still need to be externally validated (Table 2) [71]. Therefore, the discussion focuses on RV involvement.

CMR represents the gold-standard imaging technique in patients with a suspected diagnosis of ARVC, allowing for the evaluation of RV volume, morphology, mass, thickness, and wall motion abnormalities (RV regional akinesia, dyskinesia, or bulging) [70,72,73,74]. 

MRI (magnetic resonance imaging) is essential for detecting intracardiac shunts that can cause right ventricular volume overload. It is also useful in cases of suspected inflammatory conditions such as myocarditis or sarcoidosis, which may primarily affect the right side of the heart [75].

Furthermore, early regional abnormalities can be identified with CMR strain imaging, even with preserved RV global systolic function, and in some cases can predict arrhythmogenic substate in ARVC better than LGE [76,77]. 

Black-blood images are useful to identify fatty infiltration of the RV myocardium, but this finding should be used only as a confirmation tool in presence of others diagnostic criteria due to is low sensitivity [78]. 

**Figure 4:**
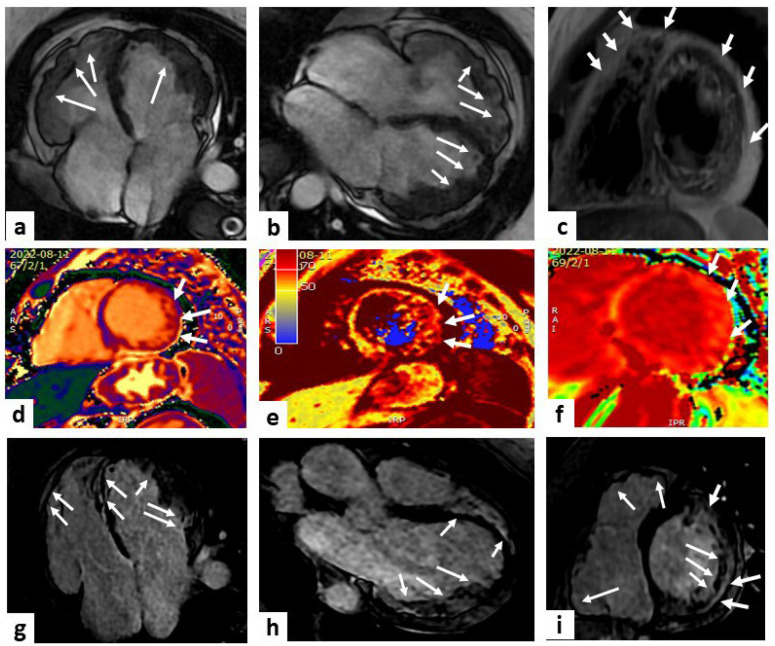
Arrhythmogenic right ventricular cardiomyopathy. A case of a 50-year-old female with a history of premature ventricular beats. Cardiac magnetic resonance (CMR) imaging showed multiple bulging of both LV and RV ventricles (white arrowheads) in steady-state free precession (SSFP) sequences (panels (**a**,**b**) show 2 slightly different four-chamber views). Possible fat infiltration of posterolateral wall emerged by proton density (PD) sequences (white arrows in panel (**c**) displaying a mid-LV short-axis view). Panels (**d**–**f**) show short-axis views of the mid-LV at T1 and T2 mapping and ECV, respectively, with evidence of a higher value at the level of the lateral wall compared to the remaining myocardium (white arrows). Late gadolinium enhancement (LGE) sequences displayed a non-ischemic hyper-enhancement pattern (white arrows) at the level of the septum, LV, and RV multiple positions (panels (**g**–**i**) show four-chamber, three-chamber, and mid-LV short-axis views, respectively). These findings are diagnostic for biventricular arrhythmogenic cardiomyopathy.

The presence and extension of fibro-fatty myocardial replacement (ARVC pathologic hallmark) can be detected using LGE imaging and is found in up to 88% of patients, with a diagnostic accuracy of 98% when wall motion alterations and pre-/post-contrast signal abnormalities were considered together [78,79,80], although LGE can be detected in other conditions (e.g., sarcoidosis, rheumatic disease, myocarditis), and its interpretation can be difficult due to RV limited thickness [81] (Figure 4).

More controversial is the role of CMR mapping as a tool for ARVC diagnosis and risk stratification. CMR mapping, recognizing areas of fibrofatty replacement in the RV, can be useful for discovering early stage disease and guide patient management [82]. In the study by Bourfiss et al., patients with genotype-positive ARVC and their at-risk family members have higher native T1 values compared to controls [83]. 

## 9. Cardiac Sarcoidosis

Sarcoidosis is an inflammatory disorder of unknown etiology characterized by the development of non-caseating granulomas that can be localized in many organs (most frequently lymphnodes, lungs, eyes, skin, nervous system), with a cardiac involvement between 27% and 80% in different autopsy series [84,85].

Cardiac sarcoidosis (CS) diagnosis remains controversial, including a proper combination of clinical signs and symptoms, ECG abnormalities, cardiac or extracardiac biopsy, and multimodal imaging (CMR, PET, CT). Two diagnostic criteria are currently used in clinical practice, one by the Japanese Circulation Society (JCS) of Sarcoidosis and Other Granulomatous Disorders and the other by the Heart Rhythm Society (HRS), both including two pathways to reach a diagnosis of CS: an histologic diagnosis, when CS is confirmed on endomyocardial biopsy, and a clinical diagnosis, when there is an histologic diagnosis of extracardiac sarcoidosis and cardiac involvement is confirmed by other findings [86,87]. 

Cardiac biopsy, despite being highly specific for the diagnosis of CS, has a poor sensitivity related to myocardial sampling errors. Cardiac non-invasive imaging, particularly CMR, has instead the ability to perform a global heart evaluation providing both functional and structural information to detect different inflammatory phases of the disease, with a sensitivity and a specificity of 93% and 85%, respectively, for diagnosing CS [88,89]. 

In the acute phase with ongoing myocardial inflammation, cine sequences are useful to visualize left and right heart contractile disfunction; regional ventricular wall thickening or thinning; and other anomalies like ventricular aneurysms, pericardial effusion, and valve pathology [88,90,91]. Moreover, signal hyperintensity on STIR T2-weighted images can describe free water accumulation due to acute inflammatory extracellular edema [92]. Global longitudinal strain abnormalities can be found in both left and right ventricles, even in asymptomatic patients, allowing for the early detection of cardiac involvement [93,94].

In the chronic phase with interstitial myocardial scarring and fibrosis, the presence of LGE, although not specific, is considered an important diagnostic parameter with a typical mid-wall or subepicardial patchy distribution in the septum, basal, and lateral wall, with possible extension to the RV [91,95,96].

Parametric mapping represents a relatively recent field of interest in CS. A few studies have shown higher values of T1 and T2 in pre-enhanced sequences (T1/T2 mapping) and in post-enhanced sequences (ECV mapping) in patients with CS compared to heathy people, even without LGE [94,97,98].

## 10. Iron Overload Cardiomyopathy

The term iron overload (IO) includes a group of disorders characterized by systemic iron accumulation and subsequent organ damage. Primary IO, known as hereditary hemochromatosis, is a genetic disease with uncontrolled intestinal iron absorption and progressive IO, while secondary IO can be caused by iatrogenic iron administration, red blood cell transfusion, hematologic conditions with ineffective erythropoiesis, or liver disease [99,100].

Historically, iron overload cardiomyopathy (IOC) has been defined as the presence of cardiac dysfunction secondary to increased deposition of iron in the heart, representing the most frequent cause of death in these patients [101,102]. Cardiac iron overload begins from the epicardium, presenting in the early stages with diastolic dysfunction and preserved LV systolic function until late phases of the disease (restrictive cardiomyopathy); subsequently, iron deposition extends to the endocardium, leading to chamber dilatation and impaired LV systolic function (dilated cardiomyopathy) [102].

**Figure 5:**
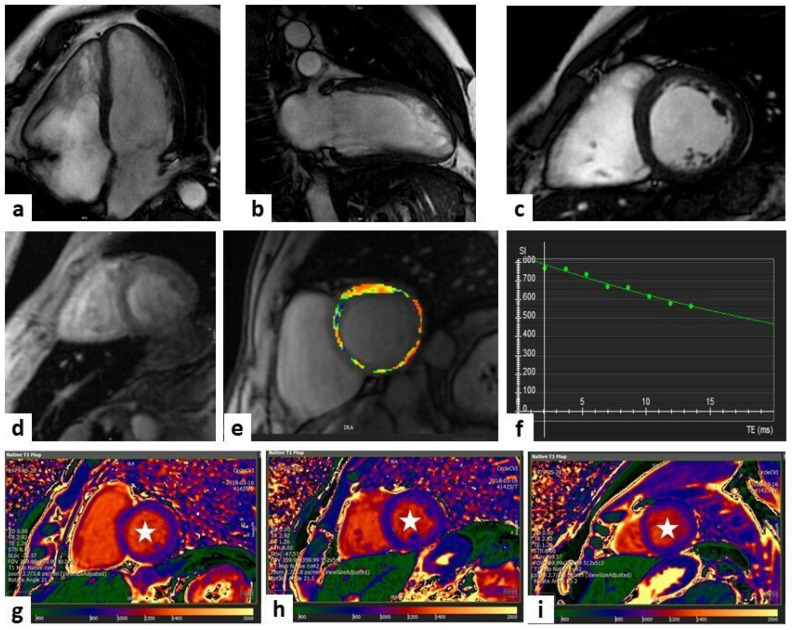
Iron overload. A case of a 38-year-old man in follow-up for thalassemia. Cardiac magnetic resonance (CMR) imaging showed a mild reduction in left ventricle ejection fraction (LVEF) in steady-state free precession (SSFP) sequences (panels (**a**–**c**) show four-chamber, two-chamber, and mid-LV short-axis views, respectively). The T2* sequences showed a normal value of iron in the heart (T2* > 20 msec), with a pathological value of iron in the liver (T2* 9 mg/g); panels (**d**–**f**) show the mid-LV short-axis view, the colorimetric display of T2* at the same level, and the graphical curve of T2* decay expressed in msec, respectively. T1 mapping showed a lower value of T1 (mean T1 900 msec), suggesting iron overload in the heart; panels (**g**–**i**) show the basal-LV short-axis, mid-LV short-axis, and apical-LV short-axis views, respectively (white stars).

CMR represents the best imaging technique to quantify myocardial IO. Due to its paramagnetic effect, iron modifies MRI signal intensity, decreasing T1, T2, and T2* relaxation times [103,104]. T2*-mapping, using gradient echo sequences, is particularly useful to identify magnetic field alterations and is currently the gold standard in IOC diagnosis [105]. A three-tier risk model for cardiac IO should be used: low risk if T2* values > 20 ms; intermediate risk if T2* values are from 10 to 20 ms, suggesting mild to moderate myocardial iron deposition; and high risk if T2* values < 10 ms, suggesting severe iron deposition [21]. Since reduced cardiac T2* is associated with an increased risk of heart failure, ventricular arrhythmias, and death, T2* monitoring has a crucial role to guide chelation therapy and assess iron overload status [106,107]. Furthermore T1- and T2-mapping can be helpful to detect patients with only mild cardiac IO, with high sensitivity and specificity [108,109]. LGE and increased ECV can be detected in patients with IO, reflecting diffuse myocardial fibrosis [110,111]. Feature tracking CMR strain imaging is a sensitive parameter for early prediction of systolic dysfunction, even in patients with normal T2* values [112] (Figure 5).

## 11. Conclusions

CMR and mapping techniques have revolutionized the diagnosis and management of cardiomyopathies by providing precise and quantitative insights into myocardial tissue characteristics. As the technology continues to advance, cardiac MRI mapping promises to further enhance our understanding of cardiomyopathies and improve patient outcomes. Its integration into routine clinical practice represents a significant step forward to treat these complex heart diseases.

## Figures and Tables

**Table 1:** CMR sequence characteristics and applications.

Sequence	Characteristics	Applications
Cine	b-SSFP sequences, cine images with high spatial and temporal resolution	Quantification of cardiac chamber size, volume, mass, and function
Black-blood imaging	T1- or PD- weighted FSE	Fatty infiltration
LGE T1-W	IR-SSFP/IR-GRE sequences, acquired after GBCA infusion	Extracellular GBCA deposition (necrosis, fibrosis, amyloid deposition)
STIR T2-W	IR-FSE sequences	Water accumulation due to inflammatory extracellular edema
Native T1-mapping	MOLLI/ShMOLLI IR-SSPF sequences	Increased in amyloid deposition, inflammatory edema, ischemia, necrosis, diffuse fibrosis; decreased in iron overload, AFD
Native T2-mapping	T2-prepared bSSFP, GraSE, FSE sequences	Increased in necrosis, ischemia, inflammatory edema; decreased in iron overload
Native T2*-mapping	GRE sequences	Decreased in iron overload
ECV-mapping	MOLLI/ShMOLLI IR- SSFP sequences, acquired after GBCA infusion	Increased in amyloid deposition, necrosis, fibrosis
FT-GLS	post-processing analysis of b-SSFP sequences with strain and strain rate deformation assessments	Assess early changes in myocardial mechanics and function

LGE T1-W: late gadolinium enhancement T-1 weighted; STIR T2-W: short tau inversion recovery T2 weighted; T2*: T2 star; ECV: extracellular volume; PD: proton density; b-SSFP: balanced steady-state free precession; IR-SSFP: inversion recovery steady-state free precession; IR-GRE: inversion recovery gradient echo; GBCA: gadolinium-based contrast agent; IR-FSE: inversion recovery fast spin echo; MOLLI: Modified Look Locker inversion recovery; ShMOLLI: Shortened Modified Look Locker inversion recovery; AFD: Anderson–Fabry disease; GraSE: gradient echo spin echo; FT-GLS: feature tracking global longitudinal strain.

**Table 2:** CMR diagnostic criteria for ARVC.

ITF Criteria (2010)		Padua Criteria (2020)	
Global or regional dysfunction and structural alterations	Major: Regional RV akinesia or dyskinesia or dyssynchronous RV contraction and 1 of the following: - Ratio of RV EDV to BSA ≥ 110 mL/m^2^ (male) or ≥100 mL/m^2^ (female) - or RV EF ≤ 40% Minor: Regional RV akinesia or dyskinesia or dyssynchronous RV contraction and 1 of the following: - Ratio of RV EDV to BSA ≥ 100 to <110 mL/m^2^ (male) or ≥90 to <100 mL/m^2^ (female) - or RV EF > 40% to ≤45%	Morpho-functional ventricular abnormalities	Major: Regional RV akinesia, dyskinesia, or bulging plus one of the following: - global RV dilatation (increase in RV EDV according to the imaging test specific nomograms) - global RV systolic dysfunction (reduction of RV EF according to the imaging test specific nomograms) Minor: Regional RV akinesia, dyskinesia, or aneurysm of the RV free wall
		Structural myocardial abnormalities	Transmural LGE (stria pattern) of ≥1 RV region(s) (inlet, outlet, and apex in 2 orthogonal views)

ITF: International Task Force; RV: right ventricle; BSA: body surface area; EDV: end-diastolic volume; EF: ejection fraction; LGE: late gadolinium enhancement.

## Data Availability

Data sharing not applicable.

## Abbreviations

ACM	arrhytmogenic cardiomyopathy
AFD	Anderson–Fabry disease
AL	light chain amyloid
ARCV	arrhythmogenic right ventricular cardiomyopathy
ATTR	transthyretin amyloid
ATTRh	hereditable transthyretin amyloid
ATTRwt	wild-type transthyretin amyloid
b-SSFP	balanced steady-state free procession
CA	sardiac amyloidosis
CAD	coronary artery disease
CHD	congenital heart disease
CMR	cardiac magnetic resonance
CS	cardiac sarcoidosis
CT	computed tomography
CRT	cardiac resynchronization therapy
DCM	dilated cardiomyopathy
DSP	desmoplakin
EBM	endomyocardial biopsy
ECV	extracellular volume fraction
ESC	European Society of Cardiology
FLNC	filamin C
FT	feature-tracking
GBCA	gadolinium-based contrast agent
HCM	hypertrophic cardiomyopthy
HRS	Heart Rhythm Society
ICD	implantable cardioverter defibrillator
IO	iron overload
IOC	iron overload cardiomyopathy
ITF	International Task Force
JCS	Japanese Circulation Society
LGE	late gadolinium enhancement
LV	left ventricle
MACE	major adverse cardiac events
MRI	magnetic resonance imaging
n-T1	native T1 mapping
NDLVC	non-dilated left ventricular cardiomyopathy
PET	positron emission tomography
QALE	query amyloid late enhancement
RV	right ventricle
STIR	short tau inversion recovery
T1-W	T1-weighted
T2-W	T2-weighted
